# The Cellular Composition of the Uveal Immune Environment

**DOI:** 10.3389/fmed.2021.721953

**Published:** 2021-10-29

**Authors:** Ian R. Reekie, Srilakshmi Sharma, Andrew Foers, Jonathan Sherlock, Mark C. Coles, Andrew D. Dick, Alastair K. Denniston, Christopher D. Buckley

**Affiliations:** ^1^The Kennedy Institute of Rheumatology, University of Oxford, Oxford, United Kingdom; ^2^Oxford University Hospitals National Health Service (NHS) Foundation Trust, Oxford Eye Hospital, Oxford, United Kingdom; ^3^School of Clinical Sciences, University of Bristol, Bristol, United Kingdom; ^4^National Institute for Health Research Biomedical Research Centre, Institute of Ophthalmology, Moorfields Eye Hospital, University College London, London, United Kingdom; ^5^Institute for Inflammation and Ageing, College of Medical and Dental Sciences, Queen Elizabeth Hospital, University of Birmingham, Birmingham, United Kingdom

**Keywords:** uvea, uveitis, iris, ciliary body, choroid

## Abstract

The uveal tract consists of the iris, the ciliary body and the choroid; these three distinct tissues form a continuous layer within the eye. Uveitis refers to inflammation of any region of the uveal tract. Despite being grouped together anatomically, the iris, ciliary body and choroid are distinct functionally, and inflammatory diseases may affect only one part and not the others. Cellular structure of tissues direct their function, and understanding the cellular basis of the immune environment of a tissue in health, the “steady state” on which the perturbations of disease are superimposed, is vital to understanding the pathogenesis of those diseases. A contemporary understanding of the immune system accepts that haematopoietic and yolk sac derived leukocytes, though vital, are not the only players of importance. An array of stromal cells, connective tissue cells such as fibroblasts and endothelial cells, may also have a role in the inflammatory reaction seen in several immune-mediated diseases. In this review we summarise what is known about the cellular composition of the uveal tract and the roles these disparate cell types have to play in immune homeostasis. We also discuss some unanswered questions surrounding the constituents of the resident leukocyte population of the different uveal tissues, and we look ahead to the new understanding that modern investigative techniques such as single cell transcriptomics, multi-omic data integration and highly-multiplexed imaging techniques may bring to the study of the uvea and uveitis, as they already have to other immune mediated inflammatory diseases.

## Introduction

**Figure 1:**
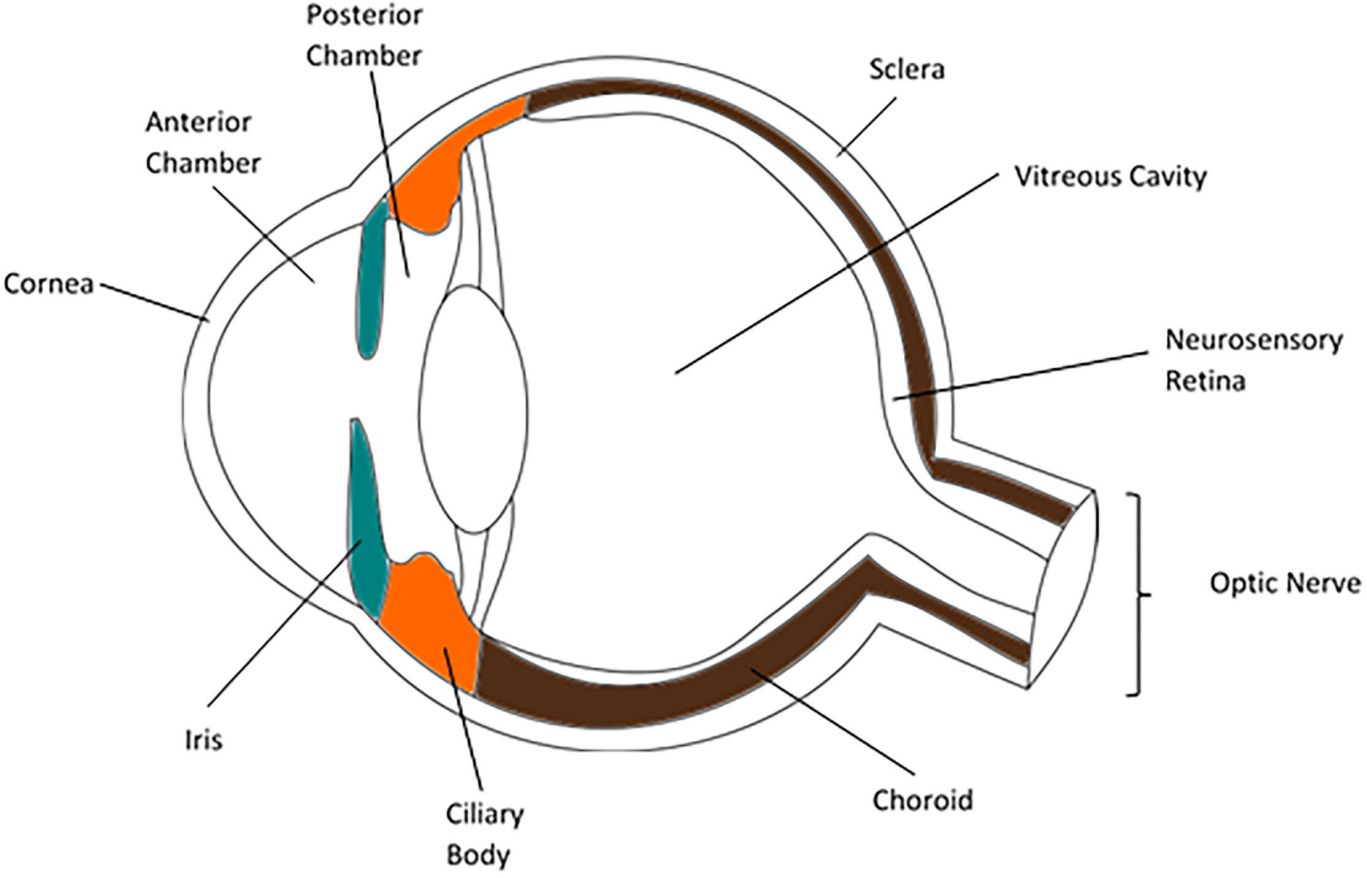
Diagram of a cross section of an eye, highlighting the iris (blue), ciliary body (orange) and choroid (brown).

The eye has several functions that make it unique amongst organs. It is required to be optically clear along the visual axis to allow light to pass through with minimal interruption, to focus light reflected or produced from sources at variable distances, and to transduce information carried by light into neural impulses to be transmitted to the brain for integration and spatial interpretation. The anatomy of the globe reflects these functions. The cornea and crystalline lens refract light through the transparent aqueous and vitreous and onto the neurosensory retina, where it is detected by photopigments and neural impulses are generated. The uveal tract is the middle vascular and pigmented layer of the eye and is comprised of the iris, ciliary body and choroid. These structures have a range of functions; forming the aperture through which light is focused, producing the internal fluids of the eye, absorbing unwanted light from outside or reflected within the eye and providing arterial supply and venous drainage to other layers of the globe (Figure 1). Anatomical lymphatic vessels have not been convincingly described in the healthy uvea in most animals studied, though they are found in the conjunctiva (1).

The uvea is the focus for a group of diseases falling under the umbrella term of uveitis. Inflammation within the eye may profoundly interrupt its key functions, causing impairment of vision alongside other well-documented symptoms of inflammation. Clinical classification of uveitis is anatomical, describing the area of the eye affected. Anterior uveitis is confined to the anterior chamber, intermediate uveitis mainly affects the vitreous and posterior uveitis the choroid or retina. Panuveitis describes a case affecting each of these areas (2). Clinically distinct conditions cause uveitis at different sites, and inflammation may extend into adjacent tissues.

The immunological environment of the eye is endowed with highly tuned regulatory immunological properties and thus recognised as an immune privileged site. By this, it is meant that circumstances that would usually lead to marked inflammatory reactions at other body sites do not produce such damaging effects, which allows the core visual function of the eye to continue (3–5). Despite ocular immune privilege however, uveitis is not uncommon and is a major cause of blindness worldwide (6). Historically inflammation has been thought of as a process driven mainly by leukocytes, but it is now acknowledged that the stroma of an affected organ also has a vital role to play in inflammatory conditions. Stroma is an umbrella term for tissue types traditionally seen as fulfilling a structural role for organs, distinct from the parenchyma that provides a specific function unique to that organ. Far from being inactive bystanders however, stromal cells are often active players in inflammatory conditions, and may take on distinct phenotypes in inflamed tissues (7, 8).

The uveo-centric nature of many ocular inflammatory diseases makes an understanding of the basic cellular constituents of the uveal immune system fundamental to a wider understanding of ocular inflammation and infection. This review discusses what is known about the immune environment of the three anatomical structures that make up the uveal tract, both in terms of leukocytes, classical immune cells, and the wider stromal cellular population. We will focus predominantly on the healthy eye, the resting state over which inflammatory states are superimposed in disease.

## Iris

**Figure 2:**
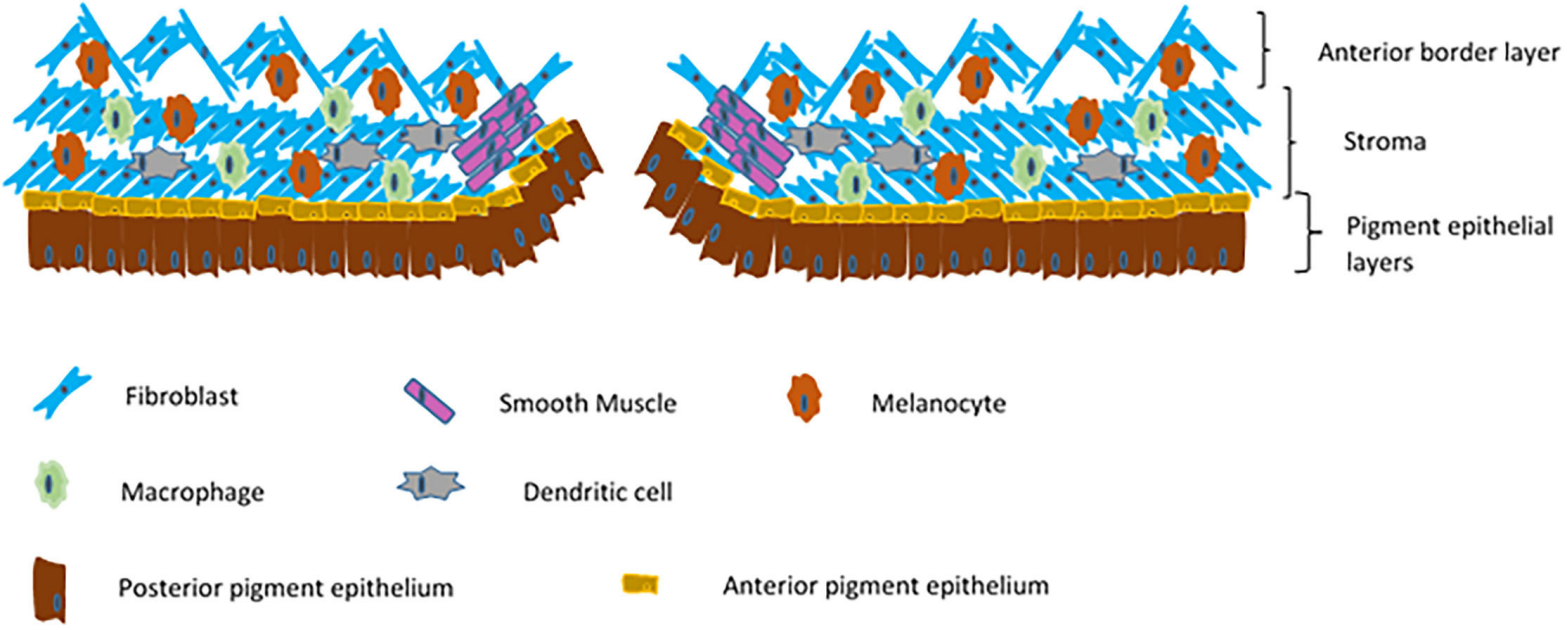
Schematic of a cross section of an iris.

The iris is a contractile diaphragm of tissue with a central aperture, the pupil. It separates the anterior from posterior chambers of the eye and is continuous peripherally with the ciliary body and trabecular meshwork. From anterior to posterior its constituents are the anterior border layer, the stroma, the anterior iris myoepithelium which makes up the dilator pupillae muscle, and the posterior pigment epithelium. The anterior border layer is discontinuous and made up of fibroblasts and melanocytes along with collagen fibrils. Defects in this layer allow contact between the aqueous and the iris stroma, which also consists mainly of collagens, fibroblasts and melanocytes. Within the iris stroma sits the sphincter pupillae muscle, the pupil constrictor. This smooth muscle structure lies close to the pupillary margin. The cells of the anterior pigment myoepithelium are arranged with their apices pointing posteriorly, the anterior basal portions of the cells have contractile elements producing the dilator pupillae muscle. The apices of these cells are pigmented and abut the apices of the darkly pigmented posterior pigment epithelium (Figure 2).

The leukocyte population of the healthy iris is, to our current knowledge, largely composed of cells of the myeloid lineage. However, much of the work that has been undertaken to identify the immune cell population of healthy eye tissue has been performed in rodents; the eyes of Lewis rats have been widely studied due to their common use for the Experimental Autoimmune Uveitis (EAU) model of uveitis (9). Dendritic cells are the prototypical antigen presenting cell, and are frequently seen in sections and whole mounts of Lewis rat iris (10, 11), as well as those of other rat strains (12), as defined by the presence of MHC class II molecules on their surface, dendritiform morphology and lack of staining for macrophage markers. Dendritic cells are similarly seen within the iris of mice and are thought to be functionally immature, lacking the co-stimulatory molecules CD80 and CD86 required to activate a T-cell response (13). It is known that freshly isolated antigen presenting cells from the mouse iris lack the ability to trigger lymphocyte proliferation *in vitro* (14), though they may gain this ability when they receive maturation signals, as rat iris dendritic cells do when cultured with GM-CSF (15). A study using bone marrow chimeric rats showed that the dendritic cell population of the iris has a residence half-life of 3 days, and replenishment of dendritic cells is a bone marrow dependant process, consistent with other tissues (16).

Some authors have advocated pig eyes as being more similar to human eyes than those of rodents, and the porcine iris also contain numerous dendritic cells, in addition to other myeloid lineage cells (17). Dendritic cells are also widespread in the human iris (18). A subset of dendritic cells in human iris tissue have been shown to express the Toll-Like Receptor (TLR) 4 complex, including MD2 and CD14 which is involved in response to lipopolysaccharide. These have a largely perivascular niche, as well as being more common in the iris root than elsewhere in the iris (19). Dendritic cells are now commonly sub-classified as conventional (or myeloid), or plasmacytoid dendritic cells, and each of these subtypes is specialised in defence against specific pathogen types or danger signals (20, 21). Dendritic cells within the uveal tract as a whole have been found to be myeloid dendritic cells, no plasmacytoid dendritic cells have so far been identified (22).

In Lewis rat iris tissue, resident macrophages are widespread (10, 11) and commonly have a perivascular niche, predominantly along the arterial circulation, as well as a perineural distribution (23). This is in contrast to the dendritic cells also identified in the same sections which were more evenly spaced without apparent preference for perivascular or perineural areas. The morphology of the rat iris macrophages documented by McMenamin et al. was variable. Macrophages within the rat iris have a slower turnover rate than dendritic cells in the same tissue, and resupply is also bone marrow dependant (16) indicating that at least a portion of these cells derive from the monocyte-macrophage lineage rather than being primitive yolk-sac derived tissue resident macrophages (24). Mouse irises have a density of tissue macrophages similar to that of rats (13), though it is acknowledged that there are strain based differences in immune cell numbers within the same species. Recent work with the C57BL6/J mouse strain identified three subtypes of tissue macrophage within the iris, defined by surface markers on flow cytometry as MCH II^−^ (69% of iris macrophages), MHC II ^+^ CD11c^−^ (23%), and MHC II ^+^ CD11c^+^ (8%). Lineage tracing using a tamoxifen inducible Cre recombinase to force Green Fluorescent Protein (GFP) production in Cx3cr1+ cells suggested the MCH II^−^ population to be largely long lived self-replenishing tissue macrophages, while the two MCH II^+^ populations were majority circulating monocyte derived macrophages. These lineage tracing experiments, however, examined single cell suspensions of whole eyes, rather than the iris alone (25). Work from other groups using similar methods has also shown that the murine iris contains both long lived tissue macrophages and short lived, presumed monocyte derived, macrophages (26).

Pig irises, in common with those of rodents, also contain macrophages which are positive for CD163. The healthy human iris stroma has been found to contain a network of tissue macrophages similar to that of rodent irises (23), though heavier pigmentation makes study of human tissues by microscopy more challenging. Further work corroborated these findings, locating macrophages as defined by staining with the PM-2K monoclonal antibody (27), a marker that is believed to label tissue macrophages specifically (28). Healthy porcine irises examined as wholemounts contain no granulocytes (17), a finding which is thought to hold across species (27), though work looking at granulocytes in healthy eye tissue is rare.

Mast cells are also of the myeloid lineage, and are present in tissues throughout the body. Mast cells are rare in the rodent iris (27, 29). Cook and colleagues have reviewed the role of mast cells in ocular disease, including a discussion on the relative distribution of mast cells throughout ocular tissues in different animals, which does vary widely (30). This is a helpful reminder that immunological differences are important to consider when extrapolating data generated from animal experiments to humans. In the human iris, mast cells are present, though they are fewer in number than in other regions of the uvea such as the choroid (31). Human mast cells are classified based on the protease content of the granules present in the cytoplasm which are released on activation; they may contain both tryptase and chymotryptase (TC mast cells) or tryptase alone (T mast cells), and these cell subtypes have distinct relative tissue distributions (32). A third minor population containing chymase only is also described (33). Within the normal human iris stroma TC mast cells are by far the most prevalent and have a scattered distribution, while mast cells of the sphincter pupillae muscle are largely of the tryptase containing subtype. Interestingly T mast cells are also the dominant subtype in the ciliary muscle (31). The relative dearth of mast cells in the iris compared to the choroid has been hypothesised to relate to the lack of capillary fenestrations in the iris vessels (31).

It is widely thought that the uvea as a whole is entirely devoid of lymphocytes, outside of circulating cells in blood vessels (3). However, there are occasional references to lymphocytes in the healthy iris. In Lewis rats T-cells may be seen very rarely in the normal iris (10, 11), within the rat iris the rare T-cells are of the αβ T-cell receptor type, with no γδ T-cells apparent (16). Occasional T-cells have been identified in pig iris wholemounts, though B-cells are noted to be absent (17). Normal human iris biopsies have also been reported to contain occasional T-lymphocytes, but not B-lymphocytes or plasma cells (34). Indeed B-cells are not found in normal uveal tissue in any species (27). Over the last decade interest in, and understanding of, tissue resident lymphocytes has grown markedly (35) and Resident Memory T-cells (T_RM_) have been located in several tissues (36, 37). Other lymphocyte classes such as Innate Lymphoid Cells (ILCs), and non-classical TCR expressing T-cells, for example γδ T-cells, are commonly tissue resident (36). It is possible then that the rare description of T-lymphocytes within the uvea represent one of these populations, with the caveat that circulating blood contamination must be carefully considered as a possible source.

The vast majority of the iris tissue mass is made up of structural cell types, such as epithelial cells of the anterior and posterior epithelial layers, fibroblasts of the stroma and melanocytes, which are present in considerable numbers in the human iris and throughout the rest of the uveal tract. Traditionally considered to be involved only in protection from the effects of the ultraviolet frequencies of the electromagnetic spectrum, melanocytes are increasingly acknowledged to have more diverse roles (38). Melanocytes in other tissues have been noted to constitutively express numerous TLRs, which have been shown to be functional upon presentation of the appropriate Pathogen Associated Molecular Patterns (PAMP) (39, 40). There is evidence that skin melanocytes respond via TLRs to some pathogens (41) and melanocytes in the iris also respond to Lipopolysaccharide (LPS) stimulation by producing IL-8 and MCP-1 (42). Furthermore, treating cultured uveal melanocytes with IL-1β causes an increase in IL-6 production, partially mediated by the NF-κB transcription factor (43). Additionally, uveal melanocytes including those from the iris, constitutively produce low levels of Matrix Metalloproteinase 8 (MMP8) and increase production under control of TNFα (44). MMP8 has a role in tissue remodelling and inflammation, and is best known as an extracellular protease produced by neutrophils. It is also implicated in several inflammatory disease processes (45). Supportive evidence for a role for melanocytes and other ocular stromal cells in ocular immunity is that non-bone marrow derived cells appear to be capable of driving inflammation in the murine endotoxin induced uveitis model (46). Elsewhere in the body there is data supporting an immune role for melanocytes including the production of inflammatory mediators and cytokines and potentially in phagocytosis and non-professional antigen presentation (47). Some evidence exists for uveal melanocytes functioning as antigen presenting cells in disease states, but not in healthy tissue (48).

There is substantial evidence for iris melanocyte involvement in the ocular immune environment. A second pigmented cell type in the iris is the posterior pigmented epithelium, the most posterior layer of the iris which is continuous with the non-pigmented ciliary epithelium, in turn continuous with the neurosensory retina. The developmental origin of the iris posterior pigment epithelia, the ciliary body non-pigmented epithelium, and the neurosensory retina has long been thought to be from the inner layer of the embryonic optic cup (49), which itself arises from the developing forebrain and is therefore ultimately of neuroectodermal origin. More recent evidence from histological studies of human foetal tissue at different developmental ages suggests that both the anterior and posterior iris epithelium may arise from the outer layer of the optic cup, which curves inward around the rim of the cup during iris development (50). This finding requires corroboration, but it would link both iris epithelial layers with the retinal pigment epithelium (RPE), which also arises from the outer layer of the optic cup. The cells of the posterior pigment epithelium layer of the iris have also been found to have properties suggesting an immune modulatory role. Human iris pigment epithelial cells removed from post-mortem eyes within 16 h produce mRNA for the TLRs 2, 3, 4, and 6, with TLR4 mRNA showing the strongest expression, and translation of these receptors is confirmed by western blot (4). Functionality of these TLRs in aiding the eye's immune response was demonstrated by production of IL-8 and MCP-1 in response to LPS (ligand for TLR4), Pam3CSK4.3HCl (TLR1/2), Poly(I:C) (TLR3) and MALP-2 (TLR2/6). These responses were in turn blocked by TLR inhibition (51). Other components of the LPS receptor complex made up of TLR4, CD14, and MD-2 are also expressed by human iris pigment epithelial cells, which have been shown to produce IL-6 on stimulation with LPS (52). As seen with melanocytes and cells of the RPE, iris pigment epithelial cells have phagocytic capacity (53, 54), though there is no evidence of a functional role for iris epithelium phagocytosis in immunity or inflammation.

The iris pigment epithelial contribution to the ocular immune system extends to a postulated role in ocular immune privilege. Mouse iris pigment epithelial cells can directly inhibit T-cell activation in a cell contact dependant process, measured by T-cell mitogenesis assay (55). This process appears to be at least partially dependant on CD86-CTLA-4 interactions between iris pigment epithelium and lymphocytes (56) and is not dependant on inducing apoptosis (57). In addition to direct actions on lymphocytes, iris pigment epithelium has an inhibitory effect of the ocular immune response in an indirect manner by the induction of T-regulatory lymphocytes (Tregs). CD8+, but not CD4+ T-cells are converted to a Treg phenotype by a contact dependant mechanism by mouse iris pigment epithelium (58). Notably, the TGFβ signalling pathway appears to be involved in Treg induction and Treg mediated suppression of T-cell activation (59–61). Thrombospondin 1 has also been implicated in CD8+ Treg generation by contact with iris pigment epithelium (62). Treg controlled reduction of lymphocyte activation in the mouse eye supresses several arms of the immune response, including T-helper 1 (Th1), Th2 and Th17, but different mechanisms apply to suppression of each arm. It has been shown that PD-L1 binding to PD-1 is involved with Th1 response suppression, but not Th17 response suppression (63). Pigment epithelia from other parts of the eye such as the retinal pigment epithelium possess similar capacity to induce Treg formation (64). Work with human iris pigment epithelial cells in culture has suggested that similar mechanisms are in play, with contact dependant and TGFβ dependant suppression of T-cell proliferation and IFN-γ production (65). Cultured human iris pigment epithelial cells also express PD-L1 and PD-L2, and the PD-1 pathway may be involved in control of immune responses in the human eye, as it is in the mouse (66). Clinical evidence for this comes in the form of drug related uveitis which can be triggered by checkpoint inhibitor therapy used in the treatment of a range of solid and haematological cancers (67, 68). The PD-1 pathway is commonly targeted by checkpoint inhibitors such as pembrolizumab and nivolumab which block PD-1 directly, and atezolizumab, avelumab, and durvalumab which act on PD-L1. These are prominent amongst those checkpoint inhibitors associated with several forms of uveitis, most often anterior uveitis (67, 68).

Lymphatic vessels are vital structures found throughout most of the body, they drain fluid from the interstitium into the systemic venous circulation and bring antigen presenting cells such as dendritic cells to lymph nodes to allow interaction with cells of the adaptive immune system (7). Lymphatic drainage of the conjunctiva of the ocular surface is well-characterised (69), but the internal ocular environment appears to lack lymphatic vessels (70, 71) and this property is thought to contribute to ocular immune privilege. Some iris macrophages in mice do display lymphatic markers such as LYVE-1 (69), a protein that, when expressed with podoplanin, is considered to mark lymphatic endothelial cells. A further population of LYVE-1^+^, Sca-1^+^, CD34^+^ cells were also identified in the murine iris root by the same authors and hypothesised to represent lymphatic progenitors (69). In the human iris numerous LYVE-1, CD68 double positive cells have been described, again thought to be macrophages (71). A fascinating property of bone marrow derived macrophages in the cornea has been described, which may provide the eye with functional lymphatics in times when inflammatory processes have been triggered. CD11b^+^ bone marrow derived macrophages have been observed to form tube like structures within inflamed mouse corneas, these cells also express LYVE-1, podoplanin and PROX-1 which are well-known markers of lymphatic endothelium (72). Therefore, macrophages seem capable of forming lymphatics in previously alymphatic structures when required. This has not yet been documented in the uvea, but it does represent a mechanism for providing a highly adaptive lymphatic drainage to an organ in times of need.

Notwithstanding the controversy and uncertainty around the lymphatic drainage of the internal compartments of the eye, it is clear that soluble antigens from within the eye do make their way to lymph nodes in the head and neck (73). The drainage of aqueous humour from the eye is also very well-characterised, mainly leaving the eye via the trabecular meshwork and Schlemm's canal, with a minor drainage pathway being described via the uveoscleral outflow tract. The uveoscleral, or unconventional, pathway involves aqueous humour moving directly through the tissue of the ciliary body and the perivascular spaces of the sclera (74), it is an ill-defined route as compared to the trabecular meshwork. Schlemm's canal itself is a unique structure, its endothelial cells express some lymphatic markers as well as those found on blood vascular endothelial cells (75, 76).

## Ciliary Body

**Figure 3:**
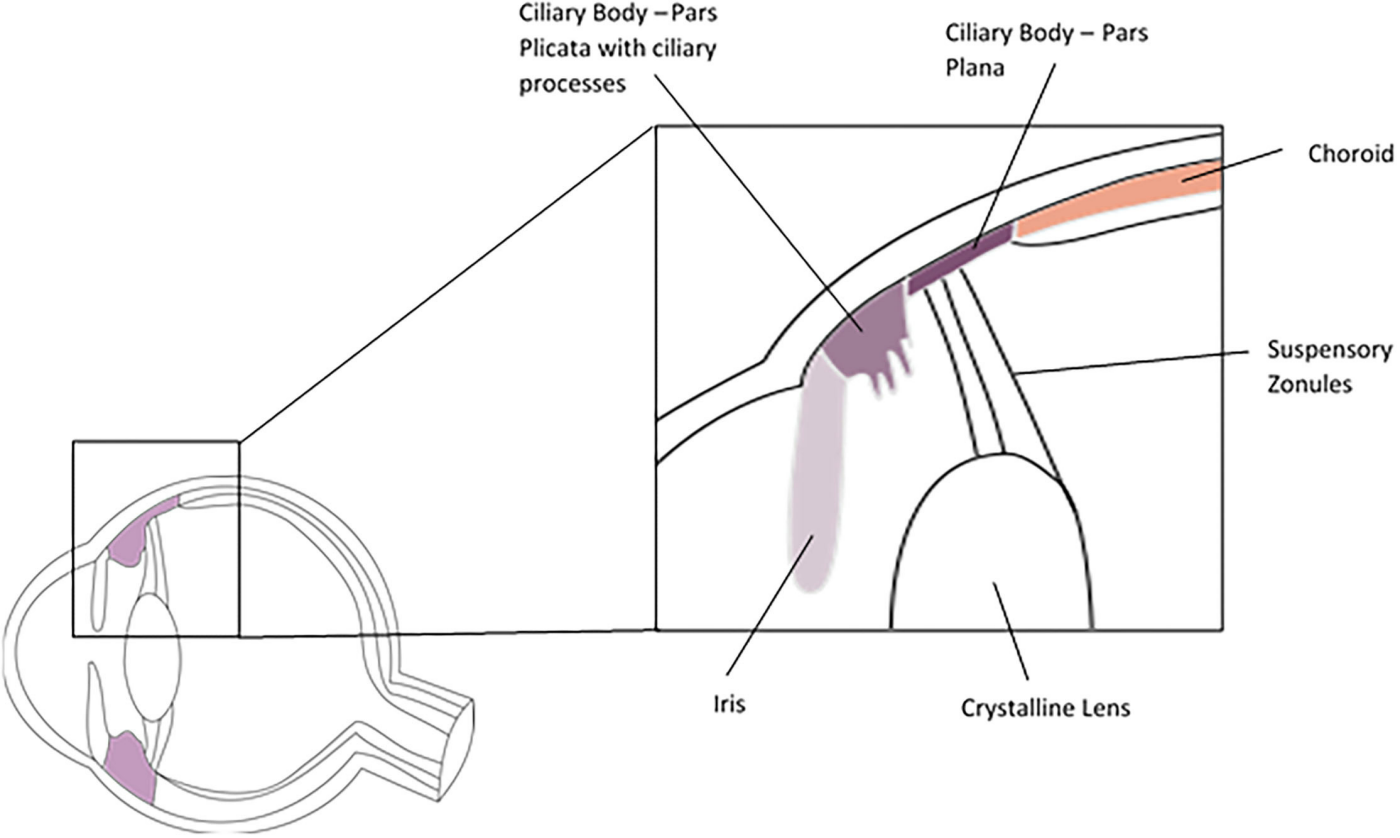
Cross section of the ciliary body.

The ciliary body is an annular structure with a triangular cross section. It is continuous with the iris anteriorly and the choroid posteriorly and is divided into the pars plicata anteriorly and pars plana posteriorly (Figure 3). The pars plicata is formed into the ciliary processes, which produce the aqueous humour. The pars plana is posterior to the par plicata and extends to the ora serrata, where the neurosensory retina begins. Histologically the ciliary body epithelium consists of the pigmented and non-pigmented epithelia, which form a bilayer on the inner surface of the ciliary body facing the aqueous and vitreous, the stroma and the ciliary muscle. The inner non-pigmented ciliary epithelium is in direct contact with the aqueous and vitreous, non-pigmented epithelial cells of the pars plicata produces aqueous by active secretion and the cells are held together by tight junctions. The non-pigmented epithelial cells of the pars plana are also secretory and synthesise the vitreal collagens and the suspensory zonules (of Zinn) of the crystalline lens, which are primarily comprised of fibrillin and pass from the pars plana to insert into the lens capsule. The outer pigmented ciliary epithelium is continuous with the retinal pigment epithelium at the ora serrata. The stroma of the ciliary body comprises fibroblasts, melanocytes and occasional leukocytes. Within the stroma sits the ciliary muscle which is a smooth muscle structure allowing accommodation when the eye focuses on near objects. Due to its close anatomical association with the iris, the ciliary body is often considered in conjunction with the iris in studies of its cellular composition (77).

The presence of dendritic cells and macrophages is well-documented within the ciliary body, as with the iris. Dendritic cells within the ciliary body of the rat have been noted to sit with the cell bodies between the pigmented and non-pigmented ciliary epithelium (12). Tissue macrophages are less common than dendritic cells in the epithelial layers, but are seen frequently in the underlying lamina propria on which the epithelial layers sit (12) and are also found in large numbers in the ciliary body stroma from where they are seen to follow the course of blood vessels to the ciliary processes (23). Within the ciliary body of the mouse, the stroma contains large numbers of tissue macrophages, and as with the rat dendritic cells these are found in the plane of the epithelial layers. Dendritic cells within healthy mouse uveal tissue appear to lack the necessary co-stimulatory molecules to activate T-lymphocytes, suggesting that further maturation is required prior to antigen presentation (13). Similar to the iris, the murine ciliary body contains both long lived tissue resident macrophages and shorter lived, monocyte replenished macrophages (26). Comparatively little work has been done on the immune cell composition of the human ciliary body, even in comparison to other areas of the uveal tract, but from what data is available it appears that the distribution of macrophages and dendritic cells is similar between rodent and human ciliary bodies (23). A small study found numerous CD163^+^ macrophages in the stroma of the healthy human ciliary body, while CD68^+^ were absent (78). This has been used to support the theory that, under homeostatic conditions, the ciliary body microenvironment is “M2” polarised in terms of the M1 vs. M2 macrophage paradigm, although making this determination on the basis of CD68 and CD163 alone has been challenged (79).

Mast cells are similarly rare in the rodent ciliary body and iris (80) but where present are mostly found in the stroma of the ciliary body proper and the ciliary processes (27). In the human ciliary body, the microanatomical distribution of mast cells is quite diverse. The stroma and ciliary muscle has a higher density of mast cells than the iris, and these are split between a TC mast cell dominant population in the stroma and a T mast cell dominant ciliary muscle (31). The ciliary processes have a very low density of mast cells, but those that are present are almost exclusively TC mast cells (31). The healthy ciliary body contains very few, if any, T-cells and no B-cells when examined by immunohistological techniques (14), though these may fail to detect rare cell populations.

The stroma of the ciliary body is similar in cellular composition to that of the iris, and as with studies on the immune cells of the ciliary body much of the work on the ciliary body stroma is done in conjunction with iris tissue. Ciliary body melanocytes have similar properties to those from the iris in terms of constitutive and induced expression of inflammatory cytokines and other mediators (42–44). The stromal immune environment of the ciliary body does, however, have some differences to that of the iris. The ciliary body pigment epithelium, unlike that of the iris, does not seem to have the capacity to induce Treg formation (58). In this regard it also differs from the retinal pigment epithelium, which has also shown Treg induction capacity (81). Ciliary body pigment epithelium does have an immune regulatory role, which while involving cell-cell contact (57) is less dependent on contact than that of the iris pigment epithelium (55) and involves secretion of immune mediators (62) including TGFβ (82). These findings support the hypothesis of an immune skewing “educational gate” element to the blood aqueous barrier (83), which is formed in part by the ciliary epithelium. Another element to this barrier, analogous in many ways to the blood brain barrier, are the tight junctions between the endothelial cells of the iris vasculature.

The non-pigmented ciliary body epithelium is the innermost part of the ciliary body and is in direct contact with the internal eye microenvironment, unlike the iris non-pigmented epithelium. This layer of cells in human eyes has been found to express both TLR4 and CD14 and these are functional in response to LPS (84). Interestingly this same study makes no mention of the staining pattern of iris pigment epithelium when antibodies to TLR4 and CD14 are used, despite other studies demonstrating expression in this tissue, only mentioning staining in the iris stroma. The ciliary body, in common with the rest of the internal tissues of the eye, does not have classical lymphatic vessels, though like the iris the human ciliary body does contain macrophages positive for LYVE-1, a marker also found on lymphatic endothelium (71). This leaves open the possibility for “ducible lymphatics” as have been described in the cornea (72).

## Choroid

**Figure 4:**
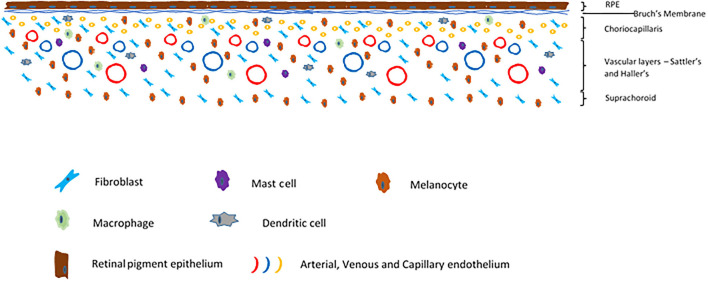
Schematic of a cross section of the choroid.

The choroid is a highly vascular, pigmented layer of tissue deep to the sclera and superficial to the neurosensory retina and retinal pigment epithelium. It is continuous anteriorly with the ciliary body. The choroid is made up of five histological layers; the inner Bruch's membrane, the choriocapillaris, the two vascular layers Sattler's and Haller's, and the outer suprachoroid which merges into the inner lamina fusca of the sclera outside it (Figure 4). Developmentally, these arise from the mesenchyme which surrounds the embryonic optic cup. Bruch's membrane is a thin connective tissue layer which itself is subdivided into five layers; the retinal pigment epithelium basement membrane, the inner collagenous layer, the middle elastic zone, the outer collagenous layer and the basement membrane of the choriocapillaris (85). The choriocapillaris is a very dense and highly anastomotic network of capillaries which supply the outer neurosensory retina. Of particular note is that the photoreceptors receive their oxygenation and nutrition from these capillaries. The choroid has among the highest blood flow by weight of any tissue (86, 87). This high blood flow keeps high oxygen saturation even in the venous blood, as even the metabolically demanding photoreceptors do not extract all available oxygen from the arterial supply (22). The capillaries of the choriocapillaris are arranged into lobules, each of which is supplied by an arteriole from Sattler's layer, which lies superficial to the choriocapillaris. Sattler's layer contains numerous arterioles and venules which are supplied from and drain to larger arteries and veins in Haller's layer, which is immediately superficial. The outermost choroidal layer is the suprachoroid which is a stromal layer primarily containing fibroblasts, melanocytes and scattered resident immune cells of the myeloid lineage, as does the stroma of the choroid as a whole. The endothelium of the choriocapillaris is fenestrated (88), in stark contact to the non-fenestrated retinal circulation which exhibit tight junctions between endothelial cells Tight junctions also exist between cells of the RPE. Thus the choroid links the eye with the systemic circulation, while the neurosensory retina has barriers which contribute to its immune privilege, akin to those in the central nervous system.

As with the iris and ciliary body, the majority of experimental data on the resident immune cell contingent of the normal choroid comes from animal models, rodent models in particular. Macrophages and dendritic cells are both found in abundance (27). Butler and colleagues utilised whole mounts of Lewis Rat choroid dissected from the retina, deep to it, and sclera, superficially, to examine the resident immune cell population by immunohistochemistry (10). They identified tissue resident macrophages based on immunostaining for CD163, known as a relatively specific marker for tissue resident macrophages (89), and found them to be widespread within the choroid and concentrated around blood vessels. Many of these resident macrophages also stained positive for sialoadhesin (90), a marker known to be present on macrophages within lymphoid organs and identified on resident macrophage populations at other sites such as the liver and lung in the rat (90). In humans, sialoadhesin (CD169) is not expressed on circulating monocytes but is on a wide range of tissue resident macrophages (91) and some activated macrophages in inflamed tissues (91, 92). In contrast to macrophages of the iris and ciliary body, which have been shown to be a mix of long-lived locally self-replenishing and monocyte derived cells, macrophages in the healthy choroid may be almost exclusively monocyte derived according to mouse experimental data (26).

Macrophages in the healthy human choroid are often found in contact with the capillaries in the choriocapillaris (93). Dendritic cells by contrast, identified based on MHC II expression, lack of staining for CD163 and their dendritiform morphology, were present at a greater density but primarily located close to the retinal pigment epithelium (10). The microanatomical localisation of dendritic cells within the choroid gave greater clarity to earlier work on rat tissue which showed their presence in perivascular tissue (94), and other studies have indicated that there are two groups of dendritic cells present which are discernible by differential levels of MHC II expression, perhaps indicating a maturation process within the tissue (95). Wholemounts of pig choroid also revealed numerous tissue resident macrophages, again with a marked perivascular preponderance, as well as dendritic cells (17). The normal human choroid outside Bruch's membrane is also populated by numerous CD68+ macrophages (96), which in the healthy state do not express inducible nitric oxide synthase, a marker of activation (97). Recent single-cell RNA sequencing data has suggested that human choroidal macrophages may be clustered into at least four subtypes based on transcriptome, with a fifth cluster primarily found in patients with neovascular age related macular degeneration (25). Two of the identified clusters with a large contribution from healthy choroidal tissue upregulated genes involved in angiogenesis compared to the other macrophage clusters, suggesting specific functional differences between macrophage subtypes within the choroid.

Mast cells have been identified and studied in the choroid of numerous species (29, 30). That they have been found within the choroid of almost all vertebrates (98) suggests a strong evolutionary drive towards their conservation. Mast cells are more numerous in the choroid than in other parts of the uveal tract in many mammals (99), including humans (31). In the rat, choroidal mast cells are more numerous towards the posterior pole of the eye than at the equator (99). Within the human choroid, the great majority of mast cells possess granules containing both tryptase and chymase and are located in the inner of the two large vascular layers. Bruch's membrane, the choriocapillaris and the suprachoroid are mostly lacking in mast cells (31). There is evidence from studies in rats that mast cells may be involved in the pathogenesis of Experimental Autoimmune Uveitis (EAU) (100), and it is interesting that different strains of rats commonly used for rodent studies show different susceptibilities to EAU which correlate to the density of mast cells found within the choroid of those strains (101).

Within the choroid, similarly to other regions of the uveal tract, it is widely held that there are no resident lymphocytes. On this point, however, experimental evidence is mixed. Some studies have found no evidence of T or B-cells within the choroid of the rat (95) or the pig (17). However, others indicate small numbers of T-cells are found within the normal choroid of Lewis rats, at a reported density of 16 ± 7 cells/mm^2^, an order of magnitude lower than the density of resident macrophages (10). Other studies corroborate these findings and, unusually, many of the T-cells identified in the choroid of both mice and humans have been found to express the γδ T-cell receptor (102), which is not the case in other parts of the uvea (16). Analysis of single cell RNA sequencing data from combined mouse retinal pigment epithelium and choroid revealed 5 immune cell clusters (103), identified by the authors as macrophages, mast cells, T-cells and two distinct dendritic cell clusters. Single cell RNA sequencing has also been performed on combined RPE-choroid of eyes from human deceased donors, though some had ocular pathology that could impact the leukocyte population present. Interestingly, in this series leukocytes clustered into B-cells, T/NK-cells, macrophages and mast cells. No mention is made of dendritic cells, which other investigators have found to make up a large part of the choroidal leukocyte population (104). It is therefore apparent that the generally held view that there are no lymphocytes within the choroid, as with other areas of the uvea, is not necessarily wholly accurate. However, if present, lymphocytes are undoubtedly less frequent within the uvea than cells of innate immune lineages.

A common theme across all three uveal sites is that stromal cells have a vital role in immune homeostasis and immune activity. This is equally true in the choroid. The choroid is an exceptionally vascular tissue (86, 87). Vascular endothelial cells have been considered in some quarters to be simply structural components of blood vessels with few other physiological roles, but it is becoming apparent that they have key roles to play in tissue and organ homeostasis and regeneration after injury (105). Within the choroid of mice, endothelial cells have a different transcriptomic profile on RNA sequencing to endothelial cells from other organs (103). The Indian Hedgehog (IHH) gene, encoding a member of the hedgehog family, is more highly expressed in choroidal endothelial cells than in other locations, and downstream from this signalling molecule is a pathway that is necessary for choroidal mast cell survival. Additionally, IHH knock out animals have an exaggerated choroidal inflammatory response to NaIO_3_-induced RPE death (103). IHH expression in human choroid was also found to be higher than in comparator tissues, leaving open the possibility of a similar regulatory circuit in humans (103). Human choroidal endothelial cells also express functional TLRs 3, 4, and 9 (106), while elsewhere in the uveal tract the endothelial cells of the non-fenestrated iris capillaries are known to express components of the LPS receptor complex, which induce inflammatory cytokine production when stimulated (84). Work applying single cell transcriptomics to the human choriocapillaris also suggests age dependent changes in the transcriptome of endothelial cells. For instance, endothelial cells from older donors exhibit enriched gene expression in pathways associated with trans-endothelial leukocyte migration compared to tissue from younger individuals, with the caveat that some of the tissue from the older donor cohort came from patients with changes consistent with age related macular degeneration (107).

The human choroid is highly pigmented, as is the choroid of other primates (85). Indeed melanocytes are the most numerous cells in the choroid (108), and as with melanocytes at other uveal locations choroidal melanocytes express low levels of some cytokines constitutively, with production increasing in response to LPS (42). Choroidal melanocytes also respond to TNFα stimulation by increasing production of MMP8 (44). Cultured human choroidal melanocytes express mRNA for TLRs 1-10, and of these functional response to ligands has been demonstrated for TLRS 1-6 based on IL-8 and MCP-1 production (108). Human choroidal melanocytes also have capacity to produce a range of chemokines downstream from IFN-γ and TNF-α stimulation (109). Curiously, Jehs et al. describe a donor dependant effect of melanocytes on monocyte migration, with melanocytes from some eyes increasing migration on a transwell migration assay when stimulated with IFN-γ and TNFα, while those from other eyes decrease migration; this is not explained by an identifiable difference in secreted cytokines. Finally, choroidal melanocytes display an ability to inhibit activated T-cell proliferation, suggesting a role in immune modulation (109). It is likely that some choroidal leukocytes are impacted by interactions with the retinal pigment epithelium, given its direct contact with the innermost aspect of the choroid, Bruch's membrane, and given that choroidal dendritic cells are observed to have some processes in contact with RPE cells (27). The RPE, like the pigment epithelia of the iris and ciliary body, appears to have a role in immune regulation of leukocytes within the eye (4, 81) and matured dendritic cells cultured with RPE cells markedly reduce production of pro-inflammatory cytokines as well as inhibiting mixed lymphocyte reactions (110). These immunoregulatory functions, along with tight junctions between RPE cells, have led to the identification of the RPE as the outer blood retinal barrier, while the tight junctions between endothelial cells of the retinal vessels act as the inner blood retinal barrier (83, 111). It is, therefore, important to remember that the uvea has close anatomical associations with other structures with their own immunological functions.

Despite making up a large proportion of the cell population of the choroid, fibroblasts remain under-studied in this tissue. One stromal cell that has attracted growing attention on several fronts is the mesenchymal stem cell (MSC). MSCs are non-haematopoietic stem cells which have the capacity to differentiate into tissues derived from embryonic mesoderm. In an attempt to standardise nomenclature, a 2006 position statement from the International Society for Cellular Therapy established criteria for identifying MSCs, and this has been widely adopted (112). Early work on mesenchymal stem cells isolated them from bone marrow (113), but it has been possible to isolate them from numerous adult tissues, and a perivascular niche for them has been described (114). MSCs have been isolated from human choroidal tissue, and shown *in vitro* to have anti-angiogenic properties, limiting the growth of cultured choroidal vascular endothelial cells (115). Work on a mouse model has demonstrated that intravitreal administration of bone marrow derived MSCs reduced expression of numerous inflammatory mediators, including IL-1α, IL-6, iNOS and TNF, and also reduced infiltration of immune cells into the retina under inflammatory conditions (116). This is in keeping with a broader paradigm of immunoregulatory functions of MSCs, which has been reviewed elsewhere (117), and suggests a possible anti-inflammatory role for MSCs in the uvea.

Controversy surrounds the presence or absence of lymphatics within the mammalian choroid. Some evidence exists from microscopy to suggest structures with the features of lymphatic vessels (118), but more recent efforts incorporating immunostaining for lymphatic markers suggest that classical lymphatics are absent from the choroid, in the human at any rate (119). Some researchers have suggested that avian choroid does contain lymphatic vessels (120). Unusually, these are described as directly draining into choroidal veins rather than connecting to systemic lymphatics and draining into the great veins (121). Similar to the iris, murine choroid has been shown to contain a population of LYVE-1 positive macrophages (69) and a population of similar cells double positive for LYVE-1 and CD 68 has been demonstrated in human choroidal samples (119). It is clear that the question of the lymphatic drainage of the internal ocular environment has not yet been settled.

Discussion of any tissue in isolation runs the risk of creating artificial conceptual silos, which are not reflected in biology. This may be particularly true in an organ such as the eye, which has several layers distinct in structure and function and yet separated by no more than potential spaces. A discussion of the immunological environment of the choroid should therefore acknowledge its role in supporting the key function of the eye – the transduction of light to neural signal by the retina. The retina itself is a distinct immune environment, a full discussion of which is beyond the purview of this review, but its chief immune cellular player is the microglial cell (122). These long lived cells of the embryonic yolk sac derived macrophage lineage are not a homogenous population, with differences observed between populations found in the inner and outer plexiform layers (26). Tight control of microglial activation is required to avoid the damaging effects of inflammation on retinal function. This is in part achieved by microglial CD200R binding by CD200 on retinal neurons and vascular endothelium (123).

## Discussion

Histopathological study of the normal and diseased uvea in humans and experimental animals has provided a longstanding knowledgebase on the anatomical organisation of its parenchyma and stroma, and the broad outline of the cellular component of the uveal immune system is well-known. This is summarised in Tables 1–3. Myeloid lineage cells make up the large majority of the resident leukocytes, as is the case in many tissues. However, there is persisting uncertainty as to the complete leukocyte complement of the uveal niche; though some sources report T-cells within the healthy uveal tract of several animals, this is controversial. Little work has been published identifying whether these may be cells which are circulating within the blood or whether they are truly present within uveal tissues and are perhaps resident lymphocytes such as have been described in several other tissues (37). Work to elucidate the functional properties of several of the resident leukocytes of the different sectors of the uveal tract also continues. For example, the tissue macrophage populations of the uveal sub-compartments have not been fully characterised, and our understanding of these cells in the uvea is limited when compared to other tissues. Macrophages in some tissues are largely derived from the embryonic yolk sac, such as the microglia of the central nervous system (124) while those at other sites such as the lung and kidney have a contribution from haematopoietic stem cells via circulating monocytes (125). It is becoming clear that uveal macrophages are a mix of long-lived self-replicating and monocyte derived cells (25, 26), though whether the long-lived subset is ultimately embryonic yolk sac derived is not known. Functional biology of macrophages within the uveal tissues is also poorly understood when compared to many other tissues.

**Table 1:** Summary table of the immune functions of iris cell types.

**Cell**	**Immune function**	**References**
**Cells of haematopoietic origin**		
Dendritic cell (cDC subtype)	Antigen presentation, functionally immature when *in situ*	(10–16, 19, 22)
Tissue macrophage	Cytokine production and antigen presentation	(10, 11, 16, 24–27)
Mast cell	Sentinel function, immediate response to pathogens Cytokine production	(27, 29–33)
**Parenchymal and stromal cells**		
Melanocyte	Response to PAMPs, limited cytokine production	(39–44)
Iris pigment epithelium	Response to PAMPs, limited cytokine production	(51, 52)
	Inhibition of T-cell activation, induction of T-reg cells	(55, 56, 58–60, 62, 66)

**Table 2:** Summary table of the immune functions of ciliary body cell types.

**Cell**	**Immune function**	**References**
**Cells of haematopoietic origin**		
Dendritic cell	Antigen presentation	(12)
Tissue macrophage	Cytokine production and antigen presentation	(23, 26)
Mast cell	Sentinel function, immediate response to pathogens Cytokine production	(27, 31)
**Parenchymal and stromal cells**		
Melanocyte	Response to PAMPs, limited cytokine production	(42–44)
Pigmented ciliary epithelium	Immunoregulatory role	(57, 62, 82, 83)
Non-pigmented ciliary epithelium	Response to LPS	(84)

**Table 3:** Summary table of the immune functions of choroidal cell types.

**Cell**	**Immune function**	**References**
**Cells of haematopoietic origin**		
Dendritic cell (cDC subtype)	Antigen presentation	(27, 94, 95)
Tissue macrophage	Cytokine production and antigen presentation	(10, 25, 93, 96, 97)
Mast cell	Sentinel function, immediate response to pathogens Cytokine production	(29–31, 98, 99, 101)
**Parenchymal and stromal cells**		
Melanocyte	Response to PAMPs	(42, 108)
	Response to TNFα and IFNγ	(44, 109)
	Inhibition of T-cell proliferation	(109)
Vascular endothelium	Response to PAMPs	(84, 106)

Our knowledge and understanding of much of the uveal stroma is sparse. Fibroblasts make up a large proportion of the stromal cells in each part of the uveal tract, but little has been done to identify the specific properties of this cell type in the eye and within the uvea the fibroblast population remains largely uncharacterised. Within other tissues fibroblast heterogeneity is well-established, and different fibroblast populations have been shown in different microanatomical niches of the same organ. Key examples of this are the gut, where fibroblast populations vary along the crypt-villus axis (126); and the skin, where papillary and reticular dermal fibroblasts have distinct transcriptome profiles (127). The function of fibroblasts is also being revisited. It has long been accepted that these cells are involved in the production of the extracellular matrix, but the crucial role of the fibroblast to immune regulation and tissue defence is becoming increasingly apparent, and is reviewed elsewhere (128, 129). The focus of discussion here has been on cellular components of the uvea, but a significant part of the mass of the iris, ciliary body and choroid comes from the extracellular matrix, made up of a wide array of matrix proteins, the matrisome. These proteins, including collagens, proteoglycans and glycoproteins, have well-documented structural roles as well as functions in cell attachment and the binding of growth factors (130). Extracellular matrix is ubiquitous, though the precise composition differs between locales, so it is unsurprising that it has a role in host defence. Both direct antimicrobial and antiviral effects of extracellular matrix proteins, and indirect effects based on chemotactic functions towards host immune cells have been documented (131).

Recent technological advances have taken our understanding of the cellular subtypes and organisation within organs and tissues to a considerably more advanced state than was previously possible. In particular, the advent of bulk and then single cell RNA sequencing has allowed the transcriptomic phenotyping of tissues and individual cells, permitting the construction of a database of cell types defined by the genes they express. The construction of just such a database is the aim of the Human Cell Atlas project (132), which has teams working in several fields in an attempt to fully define the extent of the different cell populations that make up the human body. This project can provide the link between the known human DNA code and the individual cells that are responsible for function. Within the eye, single cell RNA sequencing has already been used to great effect in the study of the human retina (133), trabecular meshwork and uveoscleral aqueous outflow route (134, 135) and corneal limbus (136), but of the uveal tract only the choroid (in conjunction with the RPE) has so far been examined in this way (104, 107). Voigt et al. show a number of cell clusters within the combined RPE/choroid analysis, including the interesting finding of T-cells which need to be further defined. A limitation of their studies is, however, the profiling of both healthy tissue and those with dry and wet age related macular degeneration in the same analysis, at a time when then baseline transcriptome of the healthy uvea is not yet understood. Defining the cell types present in an organ by their transcriptome is only part of the task of producing an atlas. It is also vital to understand the niches within an organ which particular cell types inhabit. For this reason, the new generation of highly multiplexed immunofluorescence imaging techniques (137) which allow the localisation of tens of protein markers on a single tissue sample, along with the rapidly advancing world of spatial transcriptomics (138) will be vital in localising cellular subtypes to their microanatomical position.

Developing a more complete understanding of the immunological aspects of uveal homeostasis is not of merely academic interest. Uveitis as a spectrum of disease is a major cause of blindness (139). Worldwide, the prevalence of uveitis varies markedly across countries, as does the underlying cause (140). Defining cell populations found in different anatomical locations can shed light on localised disease processes, and many forms of uveitis affect the uvea alone (141). Of greater interest to the wider medical community are uveitides associated with systemic disease. The most common form of uveitis, acute anterior uveitis, is often, but not exclusively, associated with carriage of the HLA-B27 MHC class I allele (6), which links it to a group of conditions known as the seronegative spondyloarthropathies. Recent discoveries in this field include that of a resident T-cell in the healthy entheses of mice which may have a role in the enthesitis characteristic of many spondyloarthritides (142), a finding which was corroborated in human spinal entheses where both γδ T-cells (143) and conventional T-cells expressing a T_RM_ phenotype (144) have been found. The many extra-articular associations of the seronegative spondyloarthropathies include anterior uveitis, inflammatory bowel disease and psoriasis (145), and it is hoped that improved understanding of the biology of each location in this Joint-Eye-Skin-Gut axis will shed light on similarities that may explain these associations.

Single cell RNA sequencing has already transformed our understanding of the cellular constituents of some disease states. For example, single cell sequencing has shown that in joints affected by rheumatoid arthritis there are distinct subtypes of fibroblast making up the lining layer of the synovium and the sublining layer, and these have different contributions to disease states (146). These fibroblast groups have the same physical appearance on microscopy, and express some of the same surface markers. It is only by applying transcriptomic techniques that the differences between populations can be robustly identified. Similarly, distinct subtypes of monocytes and T-cells can be defined in rheumatoid and osteoarthritic tissue (147). The stage is therefore set for similar technologies and techniques to be employed to further our understanding of inflammatory eye disease, elucidating the leukocyte and stromal cell types and axes that drive the initiation, maintenance, and resolution of the inflammatory state seen in the uveitides. In doing this we will be able to better understand the available targets for treatment of the many forms of uveitis which cause so much visual morbidity globally.

The immunology of the uvea is a fascinating field, where leukocytes and stromal cells keep delicate balance to permit inflammatory activation where required, but maintain the immune tolerance for which the ocular environment is well-known amongst immunologists and physicians. With recently developed and emerging technologies it is clear that there is great scope to expand our understanding of both stromal and leukocyte biology in the uvea for the future benefit of the many patients who suffer from the debilitating symptoms that accompany uveitis.

## Author Contributions

IR conceived the article, performed the literature search, and drafted the article. CB conceived the article and critically revised the work. SS, AF, JS, MC, ADD, and AKD critically revised the work. All authors contributed to the article and approved the submitted version.

## Funding

MC and CB were funded through Medical Research Grant: MR/S025308/1.

## Conflict of Interest

JS is now an employee of Janssen. The remaining authors declare that the research was conducted in the absence of any commercial or financial relationships that could be construed as a potential conflict of interest.

## Publisher's Note

All claims expressed in this article are solely those of the authors and do not necessarily represent those of their affiliated organizations, or those of the publisher, the editors and the reviewers. Any product that may be evaluated in this article, or claim that may be made by its manufacturer, is not guaranteed or endorsed by the publisher.
